# An efficient way to analyze function of immune cells

**DOI:** 10.1186/2051-1426-1-S1-P253

**Published:** 2013-11-07

**Authors:** Zhong Wang, Namyong Kim, Melvin Lye

**Affiliations:** 1 Sun Yat-Sen University, Guangzhou, China; 2 Curiox Biosystems, San Carlos, CA, USA

## 

Cellular assays for suspension cells provide investigators the opportunity to interrogate the biology of cellular function of immune cells or immunotherapeutic drugs. Despite significant technical advances made during the last few years, working with suspension cells, such as primary blood cells, PBMCs (peripheral blood mononuclear cells), leukemia, myeloma, or lymphoma cells, cellular assays still suffer from limitations. For example, even for HEK 293 cells, a commonly used semi-adherent cell line, significant cell loss can occur during assay process. This loss can be further exacerbated when multi-step staining procedures are required.

We applied DropArray plates, which employ surface tension to retain cells on the plate surface, for our suspension cellular assays. For a variety of suspension cells, including PBMCs, AML cells, or B cells, efficient retention (50% to 80%) is achieved during multiple washes. Reproducibility and quality of the experimental results are improved with DropArray plates. Furthermore, the specific cytotoxic action of bispecific antibody on tumor cells can be also observed in real time.

